# Thyroid leiomyosarcoma: presentation of two cases and review of the literature^☆^

**DOI:** 10.1016/j.bjorl.2015.11.020

**Published:** 2016-03-29

**Authors:** Mehmet İlhan Şahin, Alperen Vural, İmdat Yüce, Sedat Çağlı, Kemal Deniz, Ercihan Güney

**Affiliations:** a Erciyes University KBB Klinigi, Department of Otorhinolaryngology, Kayseri, Turkey; b Erciyes University KBB Klinigi, Department of Pathology, Kayseri, Turkey

**Keywords:** Thyroid, Leiomyosarcoma, Anaplastic thyroid carcinoma, Sarcomas, Tiroide, Leiomiossarcoma, Carcinoma anaplásico da tireoide, Sarcomas

## Abstract

**Introduction:**

Leiomyosarcoma is a tumor which is rarely seen in the thyroid gland. The diagnosis may be difficult and the treatment is controversial.

**Objective:**

The objective of the study is to review the literature about a rare malignant disease of the thyroid gland which has high mortality.

**Methods:**

Two cases of thyroid leiomyosarcoma are presented and the previous 23 cases in the current literature are reviewed.

**Results:**

A total of 25 cases of thyroid leiomyosarcoma are reviewed; the most common complaint was rapidly growing anterior neck mass, and ten of the 25 patients had distant metastasis at the initial admission. Fifteen of the 25 patients died with the disease in the first 12 months after the diagnosis.

**Conclusion:**

The differential diagnosis of thyroid leiomyosarcoma is important and should be performed with other malignancies of the gland, especially with anaplastic carcinoma. The prognosis is poor and there is no consensus regarding the treatment.

## Resumo

**Introdução:**

Leiomiossarcoma é um tumor raramente observado na glândula tireoide. O diagnóstico pode ser difícil e o tratamento é controverso.

**Objetivo:**

O objetivo do estudo foi revisar a literatura sobre um tumor raro da glândula tireoide que possui alto índice de mortalidade.

**Método:**

Dois casos de leiomiossarcoma da tireoide são apresentados, e os 23 casos anteriores relatados na literatura atual foram revisados.

**Resultados:**

Um total de 25 casos de leiomiossarcoma da tireoide foi revisado. A queixa mais comum foi o rápido crescimento de um tumor cervical anterior; 10 dos 25 pacientes apresentavam metástases distantes no momento da admissão. Quinze dos 25 pacientes foram a óbito nos primeiros 12 meses após o diagnóstico.

**Conclusão:**

O diagnóstico diferencial de leiomiossarcoma da tireoide é importante e deve ser feito com outras doenças malignas da glândula, especialmente carcinoma anaplásico. O prognóstico é ruim e não há consenso em relação ao tratamento.

## Introduction

Sarcomas are an extremely rare group of tumors among all thyroid malignancies.^1^ The sarcoma types observed in the thyroid are liposarcoma, leiomyosarcoma, and angiosarcoma.^2^, ^3^, ^4^ According to the histological tumor classification of the World Health Organization (WHO), thyroid leiomyosarcoma is classified as a member of the smooth muscle tumors of thyroid glands.^5^ Up to now, leiomyosarcoma of the thyroid gland has been described in 23 cases^3^, ^6^, ^7^, ^8^, ^9^, ^10^, ^11^, ^12^, ^13^, ^14^, ^15^, ^16^, ^17^, ^18^, ^19^, ^20^, ^21^, ^22^, ^23^, ^24^, ^25^ in English literature. It is difficult to make a preoperative diagnosis of thyroid leiomyosarcoma and differentiate it from anaplastic thyroid carcinoma.^1^, ^15^ The prognosis of this tumor is poor. It has been shown that aggressive surgery, adjuvant radiotherapy, and chemotherapy have not been effective on the recurring/relapse rate or survival of the disease.^3^, ^7^, ^14^, ^15^ In this report two patients with primary thyroid leiomyosarcoma are presented with the review of the literature.

## Case 1

A 39 year old male was admitted with the complaints of weight loss and odynophagia. There was no history of a previous systemic disease. He had been smoking a pack of cigarettes per day for 20 years and consuming alcohol on a daily basis. There was no history of radiation exposure. During the physical examination, a 2-cm nodule was palpated in the left thyroid lobe. The blood count values were normal, and the patient was euthyroid.

**Figure 1:**
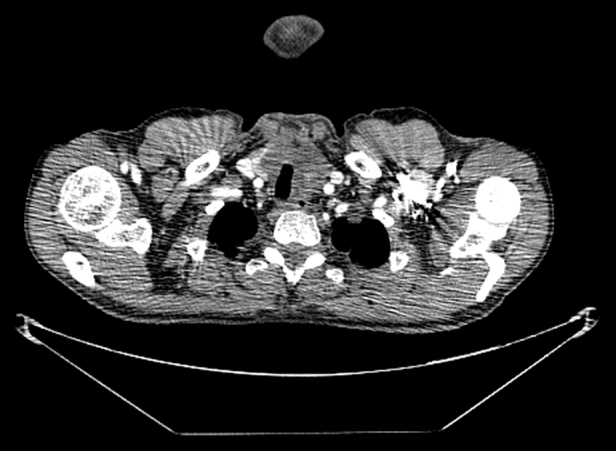
CT scan of the first patient, showing a nodular mass with dystrophic calcification.

**Figure 2:**
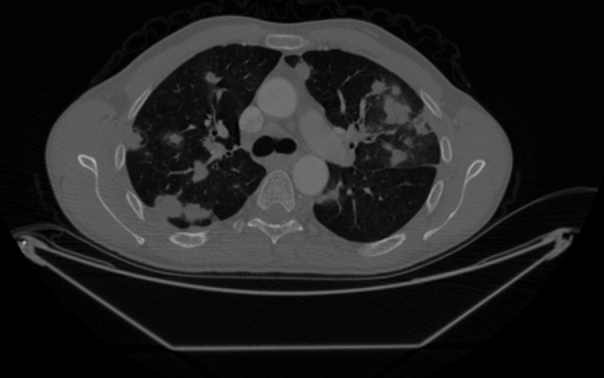
Multiple intraparenchymal and subpleural metastatic nodules in the thorax CT of the first patient.

In the thyroid ultrasonography (USG), a 24 × 26 mm hypoechoic solid mass in the left thyroid lobe was observed. Computerized tomography (CT) showed a hypodense nodular mass with dystrophic calcification in the left thyroid lobe (Fig. 1). Additionally, multiple metastatic nodules were present in the lungs (Fig. 2). A USG guided fine needle aspiration biopsy for the thyroid was not diagnostic. Upon that, surgical exploration of the thyroid bed was performed and the frozen examination from the incisional biopsy taken from the thyroid tissue yielded a malignant spindle-cell tumor.

Histological examination of the specimen showed spindle cell tumor with highly cellular fascicles. The tumor infiltrated the adjacent fat and striated muscle. 5–10 mitoses/10 HPF were counted.

**Figure 3:**
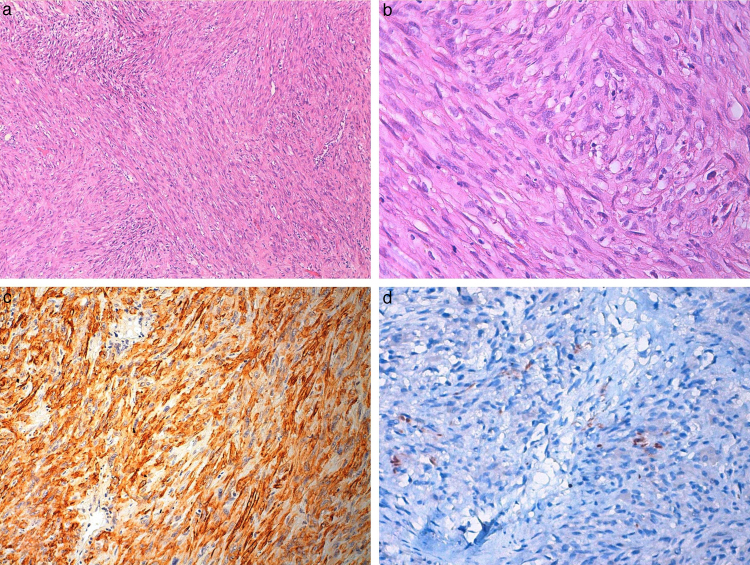
(a, b) Photomicrograph of Case 1, showing spindle cell tumor arranged in fascicular pattern with eosinophilic cytoplasm (hematoxylin and eosin ×100, ×400). (c) Tumor cells showing diffuse actin positivity (×200) and (d) focal desmin positivity (×400).

Immunohistochemical studies showed positive results for vimentin, actin, and desmin in tumor tissue, whereas other markers including pan-cytokeratin, thyroglobulin, and TTF-1 were negative (Fig. 3a–d).

Surgery was not planned because of the presence of metastatic disease. Instead, radiotherapy as a palliative treatment was planned and was applied. The patient died three months after his first visit because of diffuse pulmonary metastasis.

## Case 2

**Figure 4:**
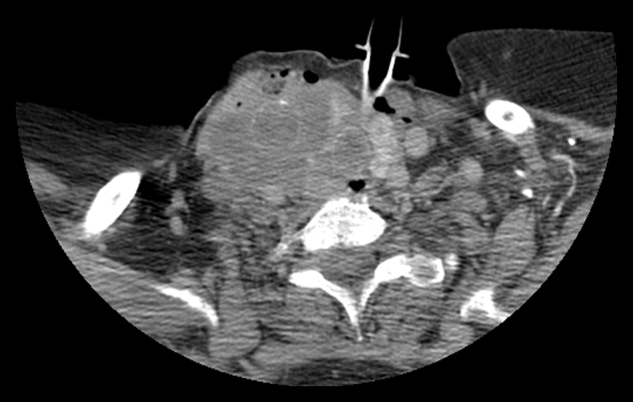
CT scan of the second patient, showing an infiltrative mass in the right thyroid lobe. Tracheotomy tube is also seen in the section.

**Figure 5:**
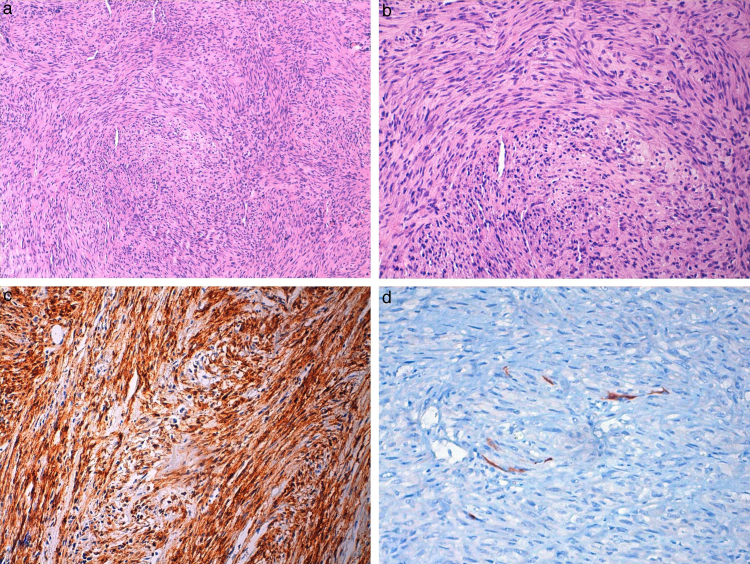
(a, b) Photomicrograph of Case 2, showing spindle cell tumor similar to Case 1 (hematoxylin and eosin ×100, ×200). (c) Tumor cells showing diffuse actin positivity (×200) and (d) focal desmin positivity (×400).

A 72 year-old female was admitted with the complaints of a rapid-growing mass in the neck and difficulty in breathing. With an aim to provide airway for the patient, tracheotomy was performed urgently. In the neck and thorax CT, an infiltrative mass was observed, originating from the right thyroid lobe (Fig. 4). The lesion spread up to the extracapsullary area, extended to the hyoid bone, and filled the third and fourth neck levels completely. It had irregular borders, and included circular calcifications. The mass surrounded and infiltrated the right common carotid artery, depressed the trachea, and narrowed the airway. On the right upper jugular area, there were multiple lymph nodes. In thorax CT, there were many metastatic nodules present in the lungs, the largest of which was 29 mm. Incisional biopsy was performed during the tracheotomy. The tumor consisted of proliferation favoring a malign mesenchymal tumor. The tumor cells had hyperchromatic nuclei and 10–15 mitoses/10 HPF were observed. Normal thyroid tissue was observed in none of the areas. Immunohistochemically, pan-keratin, thyroglobulin, and S100 were negative in tumor cells, whereas vimentin, desmin, and smooth muscle actin were found to be positive (Fig. 5a–d). Additional surgery was not conducted because the mass was evaluated as clinically and radiologically unresectable. The patient died due to impaired general condition 45 days after the diagnosis.

## Discussion

Primary sarcomas of the thyroid gland are extremely rare.^1^ Among all of the tumors of the thyroid gland, leiomyosarcoma accounts for 0.014%.^6^ Leiomyosarcoma, which belongs to the group of smooth muscle tumors, is present in 23 cases in the literature to date.^3^, ^6^, ^25^ The ages of the cases in the literature vary between 43 and 90, (except a 6-year-old patient who had immune system deficiency) and the mean age of those patients was 61.4 (Table 1). The ages of the present cases were 39 and 72 respectively.

**Table 1:** Table summarizing the 23 cases in the literature and the two patients in this report.

Reference	Age	Sex	Initial complaint	Tumor size (cm)	Lymph node metastasis	Distant metastasis	Therapy applied	Follow-up
Adachi	74	F	Mass ongoing for 4 months, pain	12	+	+	Chemotherapy	DWD, 1 month
Kawahara	82	M	A mass ongoing for 1 month, dysphonia	5.5	−	−	Lobectomy + neck dissection	DWD, 4 months, local recurrence
Kawaguchi	54	F	Mass	?	?	?	Lobectomy	Alive, 15 months, NED
Kaur	?	?	?	?	+	?	?	Alive, 12 months, LN Met.
Chetty	54	F	No symptoms	3.5	?	?	Lobectomy	Alive, 15 months, NED
Iıda	72	F	A mass ongoing for 7 months	3	−	−	Lobectomy + neck dissection	DWD, 51 months, metastatic disease
Thompson	64	F	Mass	7.5	?	+	Uncompleted surgery	DWD, 5 months, metastatic disease
Thompson	45	M	A mass ongoing for a month	9	?	+	Lobectomy, chemotherapy	Alive, 11 months, metastatic disease
Thompson	68	M	Mass, dysphonia	1.9		+	Uncompleted surgery	DWD, 18 months, metastatic disease
Thompson	83	M	Rapid growing mass, dysphagia	5.5		+	Surgery	DWD, 3 months, metastatic disease
Ozaki	58	F	Mass	5	−	−	Total thyroidectomy + neck dissection	Alive, 25 months, NED
Tulbah	6	M	Mass	5	−	+	Tumorectomy	No follow-up after 4 months, metastatic disease
Tsugawa	90	F	Breathing disorder, a mass growing in 1 month	8			Partial tumorectomy, tracheotomy	DWD, 2 months, pneumonia
Takayama	66	F	A mass which was present for 6 years that grew rapidly in 2 months	8.5	−	?	Thyroidectomy, laryngectomy	Alive, 3 months, metastatic disease, local recurrence
Day	43	M	A mass that grew in 2 months	6	−	+	Thyroidectomy, modified radical neck dissection, adjuvant imatinib mesylate chemotherapy	DWD, 6 months
Just	83	F	Mass, pain in the arm	6.7	?	−	Biopsy + palliative therapy (?)	DWD, 2 months, regional spread
Mansuri	63	F	Mass, weight loss, dysphagia	7	?	+	Total thyroidectomy	DWD, 5 months
Wang	65	F	Mass, weight loss, cough	8	−	−	Total thyroidectomy, bilateral neck dissection + chemotherapy	DWD, 4 months
Qin	–	–	–	–	–	–	–	–
Mouaqit	65	M	Left arm pain	9	−	−	Total thyroidectomy, partial esophagectomy	Alive after 5 years follow-up
Amal	72	F	Neck mass with skin fistulae	8.5	−	–	Left thyroid lobectomy with mass excision	DWD, 2 months
Tanboon	64	F	–	–	−	+	Total thyroidectomy	DWD, 3 months
Ege	56	M	Neck mass, dysphonia, dysphagia	3	−	−	Total thyroidectomy + central neck dissection	DWD, 8 months
Case 1	39	M	Weight loss, dysphagia	2.6	−	+	Biopsy + palliative radiotherapy	DWD, 3 months, metastatic disease
Case 2	72	F	Mass, breathing disorder	?	+	+	Tracheotomy + biopsy	DWD, 1.5 months

M, male; F, female; DWD, died with disease; NED, no evidence of disease.

The etiology of those tumors is unclear; however, it is thought that they develop from the smooth muscles of the veins in the thyroid gland.^3^, ^6^, ^7^ It is also claimed that leiomyosarcoma may develop as a result of smooth muscle metaplasia in a thyroid anaplastic carcinoma.^9^ The researchers who investigated the thyroid leiomyosarcoma in a 6-year-old immune deficient child claim that this tumor developed due to a malignant transformation of the smooth muscles, after being infected with Epstein–Barr virus (EBV).^12^

The initial symptom of all adult cases in the literature was mainly a rapidly growing mass in the neck. In addition, it was reported that three of the cases suffered from weight loss, four had dysphagia and odynophagia, three had dysphonia, two had pain in the arm, one had newly occurring cough, and one had breathing problems. The present report's second patient presented with rapidly growing mass in the neck and breathing problems. The first patient suffered from odynophagia and weight loss.

The initial physical examination finding was anterior neck mass in 19 of the reported 23 cases,^9^ which may be explained by rapid tumor growth. Lymph node metastasis were detected in two patients,^3^, ^8^ and an unilateral vocal cord paralysis was detected in one of these 23 patients.^7^

The review of the literature in terms of distant metastasis revealed that nine of the 23 patients had distant metastases, which were detected at the initial examinations or during follow-ups. In the present patients, lung metastasis was observed by the initial thorax CTs. Anaplastic carcinoma of the thyroid tends to cause metastasis to lymph nodes, while leiomyosarcoma rarely develops cervical metastasis.^15^

In the cases presented in the literature, the tumors observed in the ultrasonography (US) had smooth borders or were irregularly hypoechoic without halo, had cystic parts, were solid, and in some cases were calcified masses.^7^, ^9^, ^10^, ^11^ In the CTs, the masses involved large necrosis areas, with or without calcification.^6^, ^10^, ^11^ Takayama et al., who investigated the MRI findings of a case diagnosed as thyroid leiomyosarcoma, asserted that the tumor was observed to be isointense with muscle tissue in the T1 view, while it was observed with medium intensity in the T2 view. They also stated that it revealed mild signal increase in gadolinium T1.^14^ In the cases presented by Day^15^ and Just,^16^ the tumors had similar appearance in the MRI. It is realized that thyroid leiomyosarcoma is not very different from other thyroid tumors radiologically. In the first case presented in this report, a lesion in the left thyroid lobe, cystic areas, and solid nodules that include calcifications were found in the US. The CT findings revealed that this mass was hypodense and dystrophic calcifications were present in it. In the second case reported, a lesion that completely obliterated the right thyroid lobe was detected in the CT. The lesion had irregular borders and circular calcifications, and was infiltrative to the surrounding tissues.

A slight increase in the thyroid stimulating hormone (TSH) level was observed in one patient in the literature;^14^ thyroid function tests of the other reported patients were normal, and the patients presented here were also euthyroid.

An US guided fine needle aspiration biopsy revealed proliferation of atypical fusiform cells, favoring spindle cell anaplastic thyroid carcinoma, medullary thyroid carcinoma, or malignant mesenchymal tumor. With the observation of atypical fusiform cells, it was reported that in addition to medullary thyroid carcinoma and the fusiform celled version of anaplastic thyroid carcinoma, spindle epithelial tumor with thymus-like differentiation (SETTLE), solitary fibrous tumor, fibrosarcoma, and synovial sarcoma should also be considered for differential diagnosis.

The serum calcitonin levels of the patients presented in this report were within normal limits. Therefore, the possibility of medullary thyroid carcinoma vanished. However, discarding anaplastic thyroid carcinoma in the differential diagnosis is more difficult; the spindle cell type could be misdiagnosed as sarcoma.^1^ Necrosis and cystic degeneration are observed frequently in both leiomyosarcoma and anaplastic thyroid carcinomas. For exact pathological diagnosis, immunohistochemical examination and electron microscopy is very helpful,^7^, ^18^ although the latter cannot be performed routinely in daily clinical practice.

Immunohistochemically, cytokeratin staining shows epithelial origin and vimentin staining shows mesenchymal origin.^26^ While leimyosarcomas react positively with vimentin, muscle specific actin, and desmin, they do not react with keratin, thyroglobulin, chromogranin, and calcitonin.^6^ The immunocytochemical profile of anaplastic carcinoma is somewhat variable and nonspecific, although is often positive for cytokeratin.^27^ The spindle cell variant of anaplastic carcinoma can be differentiated from sarcoma with positive cytokeratin staining.^28^ In the cases presented, the fact that negative staining of the tumor with thyroglobulin, TTF-1, and keratin, and that positive staining was observed with vimentin and actin in the first case specimen, led to the immunohistopathological diagnosis of leiomyosarcoma. The second case was diagnosed with the same method as in the specimen; the tumor was negatively stained by keratin, S100, and thyroglobulin, and positively stained by vimentin, desmin, and smooth muscle actin.

Primary soft tissue leiomyosarcomas may rarely metastasize to the thyroid gland. For the patients presented in this report, the fact that there was no other focus in the radiological examinations and no previous history of surgery or malignancy discarded that possibility.

Although there are various approaches for treatment, from aggressive surgery to adjuvant radiotherapy and chemotherapy, it was reported that none of these had an effect on the recurring rate of the disease or survival.^3^, ^7^, ^14^, ^15^ The surgical approaches suggested in the literature vary from thyroid lobectomy to total thyroidectomy plus extended neck dissection. Because of the locally invasive characteristic of the tumor, some authors suggest radical surgery in order to obtain local control of the disease.^11^, ^15^, ^18^ There are also cases that had no surgical resection of the tumor, as in the above presented cases that did not undergo surgical tumor excision.

Chemotherapy is an alternative method of treatment, although it is being far from beneficial. Because of the over expression of the tyrosine kinase receptor c-kit in thyroid leiomyosarcomas, tyrosine kinase inhibitors such as imatinib mesylate are thought to be a step of the treatment approach. The patient who was treated with surgery plus imatinib mesylate died six months after diagnosis.^15^ Wang et al.^23^ presented a case with leiomyosarcoma, who underwent thyroidectomy plus anterior neck dissection and received additional treatment of ifosfamide and adriamycin, whose prognosis and outcome were not reported. Also, as an additional treatment, adjuvant radiotherapy might reduce the risk of local recurrence.^18^

The prognosis of the patients with thyroid leiomyosarcoma is poor. The disease is mostly fatal and survival rates are reported to be 5–10% in the first year.^17^ The reports in the literature indicate that 14 of the 23 patients died within months due to the disease; four of the seven who survived had a recurrent and/or metastatic disease. Among these, the longest duration of the patient to be disease-free is five years (Table). The duration between the emergence of complaints and death was a few months for both patients presented in this report.

In conclusion, although it is a rare malignancy of the thyroid, leiomyosarcoma must be taken into consideration, especially in the patients presenting with rapidly growing mass at the anterior portion of the neck and distant metastasis. Immunohistochemistry is necessary to differentiate it from other aggressive thyroid malignancies, such as anaplastic thyroid carcinoma. Leiomyosarcoma of the thyroid is an aggressive tumor and has a poor prognosis; the necessity of an aggressive oncological and surgical approach is still controversial.

## Conflicts of interest

The authors declare no conflicts of interest.
